# Comment on Liu et al. “Discrepancies of Measured SAR between Traditional and Fast Measuring Systems.” *Int. J. Environ. Res. Public Health*, 2020, *17*, 2111

**DOI:** 10.3390/ijerph17145045

**Published:** 2020-07-14

**Authors:** Mark Douglas, Niels Kuster

**Affiliations:** 1 Foundation for Research on Information Technologies in Society, 8004 Zurich, Switzerland; kuster@itis.swiss; 2 Swiss Federal Institute of Technology (ETH) Zurich, 8092 Zurich, Switzerland

**Keywords:** specific absorption rate, fast SAR measurement, field reconstruction, plane-wave expansion, traditional SAR measurement, measurement discrepancy, uncertainty analysis

## Abstract

An article published in the *International Journal of Environmental Research and Public Health* compares two types of specific absorption rate measurement systems—a fast system using a time-domain array and a traditional system using probe scanning. While the time-domain array system is analyzed in detail under idealized conditions, the probe-scanning system evaluation used a fixed set of scanning and evaluation parameters that are not fully compliant with the requirements of the published standards. This leads to a false comparison and the incorrect conclusion that time-domain array systems can be theoretically more accurate than probe-scanning systems. We have repeated the analysis applied in the paper using the same raw data but with state-of-the art scanning and evaluation parameters. The results confirm the high accuracy of probe-scanning systems for any field distribution. Due to the high precision, robustness, and reliability of probe-scanning systems, the results of these systems are often referred to as reference results.

## 1. Introduction

The article published in the *International Journal of Environmental Research and Public Health* by Liu et al. [1] analyzes the sources of error due to scanning and post-processing of two different specific absorption rate (SAR) measurement systems, assuming ideal calibration and mechanical construction. The first analyzed system is an array of sensors (referred to as a “fast measurement SAR system” in the paper) that uses time-domain probes and plane-wave expansion (PWE) to estimate the field in the phantom volume. This is one of the possible implementations of a measurement system, according to IEC 62209-3 [2]. The second system is the probe-scanning system of IEC/IEEE 62209-1528 [3] (referred to as “traditional SAR system” in the paper).

The paper drew our attention as it shows surprisingly high measurement errors for the probe-scanning system measurements of simple antennas. The results and conclusions are inconsistent with our own test results and with the conclusions of many other independent studies.

The authors of the article graciously shared the raw data of the antenna fields and the applied scanning parameters, such that we could repeat the analysis of their data.

## 2. Materials and Methods

The main findings from the review of the analyses of time-domain array systems and probe scanning systems are summarized in the following sections.

The eleven source antennas that were used in the analysis of Liu et al. [1] have also been used here: co-planar strip-fed monopole antennas at different frequencies on a handset-like casing (sources 1–6), vertical planar-inverted F antennas at 750 MHz and 1950 MHz (sources 7 and 8, respectively), and validation dipole antennas at 835 MHz, 900 MHz, and 1750 MHz (sources 9, 10, and 11, respectively). Each antenna is described in IEC 62209-3 Annex H and the reference functions are downloadable from IEC [2].

### 2.1. Review of the Analysis for Time-Domain Array Systems for Flat Phantoms

The article of Liu et al. addresses the inherent difficulty in evaluating the accuracy of array systems, given that they are black boxes with proprietary algorithms. The investigated time-domain array system is a two-dimensional array of non-moveable electric field probes. Therefore, the spatial resolution of the system is fixed and the system is unable to sample the field decay in the direction of propagation. The authors describe the trade-off between the sampling resolution and errors due to coupling between probes. Field distortion due to the array, which is another potentially large uncertainty source, is not addressed in the analysis of the Liu et al. article [1].

In particular, the authors investigated an idealized 2D array of time-domain sensors in a flat phantom and applied a PWE algorithm to reconstruct the SAR in the phantom volume. The authors recognized that time-domain array systems give unstable results, since the PWE algorithms are ill-conditioned due to the need to propagate the measured electromagnetic fields backwards towards the source. Filtering of the frequency content of the SAR distribution is needed to avoid instability, and the filtering coefficient (denoted as δ in [1]) cannot be known a priori and must be chosen arbitrarily. The authors selected a value of δ = 0.02 by fitting the results of the 11 source antennas. They also concede that this value gives very high measurement uncertainty for the calibration of the field magnitude and mechanical offsets of the sensors.

As explained in [1], all eleven antennas were positioned such that the SAR has decayed to zero at the edges of the array to avoid truncation errors. In practical cases, this limits the size of the wireless device that can be tested on the phantom. The errors, due to non-zero SAR at the array edges were not examined in the paper.

### 2.2. Review of the Analysis Probe-Scanning Systems

When evaluating the probe-scanning systems, the authors stated that they applied a fixed set of scanning parameters with a resolution of Δ*x* = Δ*y* = Δ*z* = 3 mm and the closest measurement points in the vertical direction at *z*_M1_ = 3 mm [4]. This set of scanning parameters is inconsistent with the minimal requirements of published standards and does not reflect the grids used by state of-the-art probe-scanning systems for more than 10 years. The three main observations are:

1. The scanning requirements of IEC 62209-1 [5] referred to in Liu et al. were already outdated, since the publication of IEC 62209-2 AMD1 in May 2019 [6], several months before the paper was submitted for publication. IEC 62209-2 AMD1 has stricter scanning requirements for sources with strong field decay. The authors also applied linear interpolation and extrapolation to some of their data despite the standard clearly recommending more accurate interpolation and extrapolation methods, such as splines and polynomials. Applying splines reduces the maximum errors to 20% compared to 40% using linear methods.
2. The high measurement errors for sources 5 and 6 (i.e., monopoles at frequencies from 5–6 GHz) in the paper by Liu et al. are due to an additional mistake in the application of the scanning parameters. The authors applied the same parameters as used at lower frequencies instead of the parameters at 5500 MHz and 5800 MHz for sources 5 and 6, respectively. Using the frequency-dependent scanning requirements would have reduced the errors from 20% to less than 4% for these two sources.
3. The authors were not aware [4] that state-of-the-art probe scanning measurement systems enable measurements as close as *z*_M1_ = 1.4 mm from the surface (i.e., much closer than the distance used in [1]). This distance has also been recommended by the manufacturer of these systems for many years. This minimizes extrapolation errors, which are a major source of measurement uncertainty for all SAR measurement systems. In addition, minimal grid steps of Δ*x* = Δ*y* = 5 mm in the plane parallel to the surface and graded grids in the z-direction for all scans are recommended. The paper of Liu et al. does not consider or mention the current practice of these state-of-the-art systems.

## 3. Results

**Figure 1:**
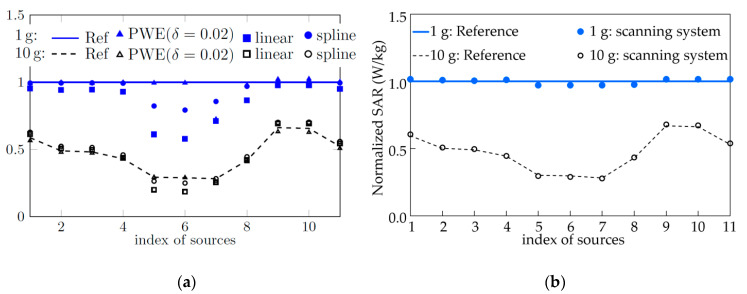
Peak 1-gram average SAR (blue) and 10-gram average SAR (black) of the eleven source antennas normalized to a target peak 1-gram average SAR of 1 W/kg. Probe scanning results are shown for linear (square markers) and spline (circle markers) methods of interpolation and extrapolation. (**a**) Results presented in Liu et al. [1]. (**b**) Results applying standard conformal grid parameters with DASY6 V6.4 [7] or later. Liu et al. [1] greatly overestimated the error of probe-scanning systems to be as much as 40%. Actual errors are less than 3%.

Figure 1b shows the results of the analysis using the same raw data with state-of-the art scanning and evaluation parameters. The actual measurement errors are less than 3% for all 11 source antennas for any lateral shift in the measurement grids with respect to the antenna. Results were generated using the post-processor engine of cDASY6 V6.4 and later using its standard EX3DV6 probes (SPEAG, Switzerland, www.speag.swiss). The results published by Liu et al. [1] are shown in Figure 1a. Table 1 compares the zoom scan parameters stated by Liu et al. [1,4] and those used by cDASY6 V6.4 applying IEC 62209-2 AMD1 for antenna 7.

## 4. Discussion

The paper of Liu et al. [1] presents an interesting analysis of the fundamental limitations of time-domain array systems and the PWE algorithms that they rely on, even though the work is limited to flat phantoms. It is expected that the errors are significantly higher for head-shaped phantoms, because reconstructing the PWE expansion for the backward propagation of waves incident on curved surfaces is much more challenging.

Unfortunately, in the comparison with the probe-scanning system, the authors made mistakes that had significant effects on the measurement errors. The scanning parameters that they used are not compliant with any published standard.

## 5. Conclusions

Measurement errors using probe scanning systems are actually less than 3% for the eleven sources, rather than up to 40% reported by Liu et al. This is also in line with the measurement experience of the IT’IS Foundation using validation sources. Probe-scanning systems have longer measurement times than array systems, yet they have the clear advantage of high accuracy for a range of phantom types due to small extrapolation errors and direct measurements in the volume of interest close to the surface of the phantom. Thus, they are often—rightly so—referred as the gold standard.

## Figures and Tables

**Table 1:** Zoom scan parameters stated by Liu et al. [1,4] versus state-of-the-art parameters expected from applying IEC 62209-2 AMD1 for antenna 7. The IEC 62209-2 AMD1 parameters are implemented in DASY6 using the EX3DV4 electric field probe [7].

Parameter	Liu et al. [1]	IEC 62209-2 AMD1
Δ*x*, Δ*y*, Δ*z*	3, 3, 3	4, 4, 1.4
*z*_M1_	3	1.4
grid in z direction	uniform	graded
graded ratio in z direction	1	1.4
